# Study on Chemical Composition and Biological Activity of *Psidium guajava* Leaf Extracts

**DOI:** 10.3390/cimb46030137

**Published:** 2024-03-06

**Authors:** Hyonam Park, Bohee Kim, Yuri Kang, Woonjung Kim

**Affiliations:** 1 Department of Cosmetic Science, Hannam University, Daejeon 305-811, Republic of Korea; westwood7456@naver.com; 2 Department of Chemistry, Hannam University, Daejeon 305-811, Republic of Korea; bohee6728@naver.com (B.K.); yuri_1005@nate.com (Y.K.)

**Keywords:** *Psidium guajava*, antimicrobial activities, tyrosinase, collagenase, trans-2-nonenal inhibition

## Abstract

Guava (*Psidium guajava*) is a plant widely distributed in tropical and subtropical regions. Its leaves contain a large amount of physiological molecules such as flavonoid, sesquiterpene, triterpenoid, coumarin, alkaloid, and tannin molecules with antioxidative and anti-inflammatory effects. In this study, the use of concentrated *P*. *guajava* leaf extract molecules as a functional natural material was evaluated by confirming the extract’s antioxidative, antibacterial, tyrosinase activity inhibition, and collagenase activity inhibition effects and its trans-2-nonenal removal ability. As a result of the analysis of the antioxidant and antibacterial components of concentrated *P*. *guajava* leaf extract molecules through GC-MS, a large amount of aromatic hydrocarbon molecules were detected. When different concentrations of ethanol were used for extraction, the leaf extract concentrated with 70% ethanol showed the most effective active molecules. As a result of measuring DPPH radical scavenging activity, a concentration-dependent antioxidant activity was confirmed. The antioxidant activity tended to increase when the ethanol content used for extraction was increased. Molecules such as 2,4-di-tert-butylphenol, caryophyllene oxide, and γ-muurolene in *P*. *guajava* leaf extract concentrate appeared to have antibacterial activities against *S*. *aureus* bacteria known to cause atopy. As ethanol content increased, the inhibitory effect on tyrosinase activity was increased. In addition, when ethanol content was 50%, the concentrated leaf extract was able to remove trans-2-nonenal by 52.4%. As a result of determining the concentrated leaf extract’s collagenase inhibition activity, an inhibition rate close to that of ascorbic acid, a positive control, was confirmed. The concentrated guajava leaf extract molecules were confirmed to have whitening and wrinkle-improving functionality. Thus, the *P*. *guajava* leaf extract has high potential as a food and natural cosmetic material.

## 1. Introduction

Recently, not only in Korea but globally, there has been a growing interest in health and skin protection. According to a report by the World Health Organization (WHO), about 80% of the population in the developing world relies on traditional plant extracts as antioxidants, antibacterial, antifungal, and antiviral agents [1,2,3]. 

In general, natural extract materials contain antioxidative molecules with various morphological properties, of which phenolic molecules are known to be representative substances with antioxidant capacity. Thus, natural antioxidants separated from them are estimated to have an anti-aging benefit [4,5,6]. Guava (*Psidium guajava*) is a dicotyledonous plant of the Myrtaceae family. It is widely distributed in tropical and subtropical regions of America. Guava can be said to be an “alternative crop that can cope with environmental changes” caused by global warming in terms of its cultivation environment and physiological characteristics. In Korea, it is known to grow mainly on Jeju Island [7]. However, due to climate change and crop technology improvements, its cultivation area is gradually being distributed widely, including inland. In addition, it is a plant that does not require separate expensive facilities. It can be considered an eco-friendly plant suitable for low-carbon, green growth. Guava fruits and leaves are high-value-added plants with great utility as functional cosmetics and food materials. They are expected to gradually expand into high-income crops.

Guava leaves have been reported to contain flavonoids, sesquiterpenes, triterpenoids, coumarins, alkaloids, and tannins [8,9,10]. In addition, studies have reported various physiological activities of concentrated *P*. *guajava* leaf extract, such as a hyperglycemia inhibitory effect [11], an antioxidant effect [12], an antibacterial effect [13], and tyrosinase inhibitory activity [14,15,16]. In particular, sesquiterpene components have been reported to have antioxidant activity, antibacterial activity, tyrosinase and collagenase inhibition activity, and trans-2-nonenal removal effects [17,18,19]. In addition, in a previous study on the physiological activity of natural extracts, it was confirmed that Cypress tree leaf extract, from which a large amount of sesquiterpene components were extracted, had an antibacterial effect of more than 80% and a high trans-2-nonenal removal activity [18]. Collagenase and tyrosinase inhibitory activity of more than 80% was confirmed in red soybean extract [17]. In wheat germ extract, tyrosinase inhibition activity of more than 80% and trans-2-nonenal removal activity of more than 80% were confirmed [19]. 

Therefore, this study aimed to determine the tyrosinase inhibitory activity, collagenase inhibitory activity, and trans-2-nonenal scavenging activity of concentrated *P*. *guajava* leaf extract, for which various bioactive molecules have been reported, to increase their value as functional natural materials and cosmetics and to secure basic data for the development of natural materials using *P*. *guajava* leaves.

## 2. Materials and Methods

### 2.1. Materials and Extraction Conditions

The *P*. *guajava* leaves used in the experiment were purchased in powder form from Jeju Island (Booyoung Oriental Pharmacopoeia, Seoul, Republic of Korea). Water and ethanol were used as solvents for immersion extraction of *P*. *guajava* leaves. Different ethanol contents (30%, 50%, and 70%) were used for extraction. The *P*. *guajava* leaf powder amount was set to be 50 g, and the total solvent was set to be 1000 mL. The immersion conditions were as follows: room temperature (24 °C) for 24 h. Extracts were filtered under reduced pressure with a 5 to 8 μm (Hyundai Micro, Seoul, Republic of Korea) filter. The filtrate was tested by removing ethanol using a rotary vacuum evaporator (EYELA N-1300, Shanghai Eyela Co., Shanghai, China), concentrating it under reduced pressure, and refrigerating it at 4 °C. In addition, extraction was performed three times to confirm the average yield.

### 2.2. Chromatic Analysis

*P*. *guajava* leaf extracts concentrated using different ethanol contents were subjected to chromatic analysis using a chromometer (CR-400, Konica Minolta, Tokyo, Japan) with lightness (L*), redness (a*), and yellowness (b*) values according to Hunter’s value. The average value was calculated after measuring each sample three times. The L* value ranges from 0 to 100, and the lower the number, the darker the color. The more positive the a* value is, the redder the color is, and the more negative it is, the greener the color is. The more positive the b* value is, the yellower the color is, and the more negative it is, the bluer the color is. All measurements were performed immediately after extraction to avoid color differences in measurement value due to oxidation. Measured values were expressed as L* (lightness), a* (redness), and b* (yellowness).

### 2.3. GC-MS Active Molecule Analysis

Gas chromatography–mass spectrometry (GC-MS) was used to analyze active molecules of concentrated *P*. *guajava* leaf extracts. HP-5ms (30 m × 250 μm × 0.25 μm) was used as a column for GC (Agilent 19091S-433, Agilent technologies, Santa Clara, CA, USA) analysis. The carrier gas used was He. The GC conditions were as follows: a temperature of 250 °C, a pressure of 7.0699 psi, and a division ratio of 10:1. The oven temperature was maintained at an initial 40 °C for 3 min and then increased to a final 325 °C for analysis.

### 2.4. Quantitative Analysis of Polyphenols and Flavonoids by LC-MS

The contents of polyphenols and flavonoids were quantitatively analyzed using liquid chromatography–mass spectrometry (LC-MS). Gallic acid (Daejeong Chemical Co., Ltd., Siheung, Republic of Korea), a polyphenol standard sample for concentrated *P*. *guajava* leaf extract, was diluted in distilled water to concentrations of 0.01, 0.05, 0.1, 0.5, 1, 10, and 100 μg/mL. It was then added to each sample diluted 10 times in distilled water. UltiMate 3000 RSLC (Thermo Fisher Scientific, Waltham, MA, USA) was used for LC, and Q-Exactive Orbitrap MS (Thermo Fisher Scientific) was used for MS. The injection volume was 5 µL. The MS condition was set to be a negative mode. Quantitative analysis was performed by setting the aux gas flow rate to 13 and the capillary temp to 350 °C. Quercetin (Sigma-Aldrich, St. Louis, MO, USA) was used as a standard sample for quantitative analysis of flavonoid content in the extract. The same equipment and analysis conditions were used for polyphenol analysis.

### 2.5. Free Radical Scavenging Ability Assay (DPPH Method)

The radical scavenging ability against DPPH (2,2-diphenyl-1-picrylhydrazyl) was determined using the reducing power of DPPH [20]. The standard used in the experiment was DPPH 95% Powder (Thermo Fisher Scientific) dissolved in methyl alcohol to form a 1.12 mM solution. 

The concentrations of *P*. *guajava* leaf extract were determined as 0 mg/mL, 0.31 mg/mL, 0.63 mg/mL, 1.25 mg/mL, 2.5 mg/mL, and 5 mg/mL, while the concentrations of control ascorbic acid were 0 mg/mL, 5 mg/mL, 10 mg/mL, 12.5 mg/mL, 25 mg/mL, and 50 mg/mL, and a standard curve was prepared and expressed as ascorbic acid equivalent antioxidant capacity (mg AEAC/g).

Then, 100 µL of the sample diluted and 900 µL of DPPH solution were mixed and reacted at room temperature for 30 min, shielded from light. The absorbance was then measured at 517 nm using a UV/Vis spectrophotometer (KLAB, Deajeon, Republic of Korea).

The DPPH scavenging activity inhibition assay was calculated using Equation (1) for the control group without adding a sample and for the experiment with a sample added:

(1) $$DPPH\;inhibition\;assay\;(\%)\;=\;\frac{{Abs}_{\left(control\right)}\;-\;{Abs}_{\left(sample\right)}}{{Abs}_{\left(control\right)}}\times 100$$

(*Abs*_(*sample*)_ = *Abs*_(*test*)_ − *Abs*_(*color*)_)

Here, *Abs*_(*control*)_ is the absorbance of DPPH, *Abs*_(*test*)_ is the absorbance of the guava leaf extract, *Abs*_(*color*)_ is the blank (ethanol) absorbance. *Abs*_(*sample*)_ represents the absorbance obtained by subtracting *Abs*_(*color*)_ from *Abs*_(*test*)_.

### 2.6. Antimicrobial Activity Measurement (Disc Diffusion Method)

For the antibacterial efficacy assay of the concentrated *P*. *guajava* leaf extract, *S. aureus* strains were cultured by disc diffusion assay [21].

Strains stored in cell stock were spread onto a solid medium and cultured in an incubator at 37 °C for 18 h. After 3 mL of liquid medium was titrated into test tubes, 1 colony of cultured bacteria was inoculated into each test tube and subcultured in an incubator at 37 °C for 18 h before use. After spreading 100 µL of liquid medium cultured on solid medium, a paper disc was placed on a weighing dish, and 40 µL of the extract was absorbed into the disc. As a positive control, discs containing 30 μg of cefotaxime and 10 μg of ampicillin were placed and incubated at 37 °C for 24 h. The size of the clear zone (mm) formed around the paper disc was measured to compare the antibacterial activity.

### 2.7. Measurement of Ability to Remove Odor of Elderly People (Fehling Reaction)

Trans-2-nonenal (Tokyo Chemical Industry, Tokyo, Japan) reagent was used to analyze the trans-2-nonenal removal efficacy of concentrated *P*. *guajava* leaf extract molecules. Cypress leaf extract was used as a positive control [18], and 100 µL of concentrated *P*. *guajava* leaf extract was added into 1 mL of trans-2-nonenal reference material diluted to 0.0075% and dispersed at 400 RPM for 30 min. After dispersion, 150 µL of Fehling Solution A and 150 µL of Fehling Solution B (Daejeong Chemical & Materials Co., Ltd., Siheung, Republic of Korea) were each injected and reacted in an oven at 50 °C for 15 min. Only the supernatant was taken and analyzed using UV/Vis. Absorbance was measured at a wavelength of 300 nm [22,23,24].

### 2.8. Tyrosinase Inhibition Activity Assay

To analyze tyrosinase inhibitory activity, which is important in measuring whitening activity as one of the important factors in the melanin production process from L-Tyrosin [25], 67 mM sodium phosphate buffer (pH 6.8), 10 mM L-DOPA (3,4-dihydroxy-L-phenylalanine, Sigma-Aldrich, St. Louis, MO, USA), and 125 units of tyrosinase (T3824-25KU, Sigma Aldrich, St. Louis, MO, USA) were prepared. After 200 µL aliquots of each substrate and sample were added to the prepared buffer and reacted in a water bath at 25 °C for 30 min, the amount of DOPA chrome produced was measured using UV/Vis at a wavelength of 475 nm [26]. The tyrosinase activity inhibition assay result was calculated using Equation (2). A control group without the addition of a sample was used. In the experimental group, a sample was added. Kojic acid was used as a positive control.

(2) $$Inhibition\;assay\;(\%)=\left(1-\frac{Experimental\;optical\;density}{Control\;group\;optical\;density}\right)\times 100$$

### 2.9. Collagenase Inhibition Activity Assay

To analyze the collagenase inhibition activity of concentrated *P*. *guajava* leaf extract molecules, a buffer (pH 7.5) was prepared by adding 1 M HCl to a mixture of 0.1 M Tris and 4 mM CaCl_2_. 4-phenylazobenzyloxycarbonyl-Pro-Leu-Gly-Pro-D-Arg (1.2 mg/mL) (Sigma Aldrich) was used as a substrate. To 50 μL of each extract sample, 75 μL of collagenase (CD130-100MG, Sigma Aldrich) at a concentration of 0.4 mg/mL and 125 μL of substrate were added. The reaction was performed in a 37 °C water bath for 30 min. After the reaction was stopped using 20% citric acid, stirring was performed at 120 RPM for 10 min. After centrifugation (HA-12, Hanil Industrial, Incheon, Republic of Korea), the supernatant was taken, and the absorbance was measured at a wavelength of 320 nm using UV/Vis [17].

The collagenase inhibition assay result was calculated using Equation (2) using the control group without the addition of a sample, the experimental group with the addition of a sample, and ascorbic acid as the positive control group.

### 2.10. Statistical Processing

In this study, all experiments were performed at least three times, and ANOVA was performed to test statistical significance according to the experimental group (*p* < 0.05).

## 3. Results and Discussion

### 3.1. Guava Leaf Extract Yield

The concentrate yields of concentrated *P*. *guajava* leaf extract according to the extraction conditions are provided in Table 1. The yield of the 30% ethanol-extracted concentrate was measured as the highest at 35.8%, while the yield of the 70% ethanol-extracted concentrate was 17.1%. The yield demonstrated a decreasing trend as the ethanol content increased. These results align with the findings of Hong et al. [27], where the extract yields increased in the order of 70% ethanol < 50% ethanol < 30% ethanol, indicating a tendency for higher yields with greater solvent polarity. This trend is consistent with the study on the antioxidant effects of quince ethanol extract by Lee et al. [28], where the yields of the fractions as a function of solvent also exhibited a pattern of higher yields with increasing solvent polarity. Since ethanol is a non-polar solvent compared to water, it is believed that the difference in polarity between the solvents leads to variations in the composition of the eluted substances.

### 3.2. Colorimeter Measurement Results

The colorimetric measurement results of concentrated *P*. *guajava* leaf extract are shown in Table 2. The luminosity, represented by the L* value, was highest at 20.9 ± 0.56 in the 30% ethanol extract and lowest at 11.8 ± 0.25 in the 70% ethanol extract. The values for redness (a*) and yellowness (b*) also showed a decreasing trend as the ethanol concentration increased from 30% to 70%.

### 3.3. GC-MS Active Molecule Analysis Results

**Figure 1:**
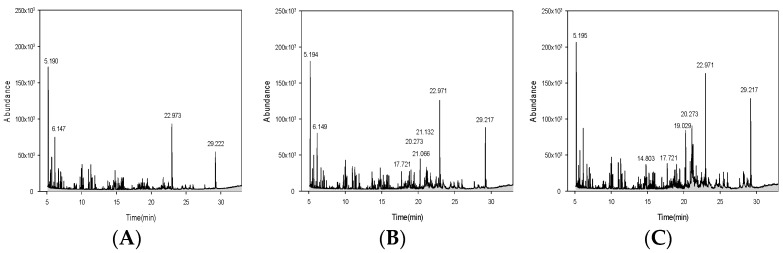
GC-MS chromatograms of extract of *Psidium guajava* leaves: (**A**) 30% ethanol extract, (**B**) 50% ethanol extract, (**C**) 70% ethanol extract.

As a result of GC-MS analysis, large amounts of antioxidant and antibacterial molecules were detected in concentrated *P*. *guajava* leaf extract. When leaf extracts using different ethanol contents were compared, the extract using ethanol content of 70% had the most active molecules. The results are shown in Figure 1. As shown in Figure 1, among the many ingredients, those with antioxidant, antibacterial, tyrosinase, and collagenase activities were indicated based on previous research results [17,18,19]. In particular, of these different molecules, 1-octadecene and 1-docosene are substances known to have antioxidant and antibacterial properties. In guava leaf extracts extracted with 30%, 50%, and 70% ethanol, 1-octadecene and 1-docosene were at 22 min and 29 min. Such a high content of these molecules indicates that guava leaf extract has a strong antioxidant activity with a high potential for use as a natural antioxidant material. Also, active molecules of guava leaf extract, such as caryophyllene and copaene, are sesquiterpene components that can inhibit the activity of tyrosinase, thereby reducing the oxidation reaction of tyrosine through tyrosinase and inhibiting melamine formation [29,30].

The results of comparing effective active molecules according to ethanol content used to extract guava leaves are shown in Table 3, Table 4 and Table 5. A large amount of aromatic hydrocarbon molecules was detected in all extracts. In addition, sesquiterpene components were increased with the increase in the ethanol content used for extraction. In Figure 1, the 1-docosene component appears to be highest in the 50% ethanol extract, but looking at Table 3, Table 4 and Table 5, it can be seen that the area peaks (%) are similar. It is well known that 1-docosene possesses antibacterial and antioxidant effects. It can be seen that these molecules have antibacterial and antioxidant effects in guava leaf extracts extracted with 30%, 50%, and 70% ethanol. Therefore, compared to previous research results, ingredients extracted from guava leaves show high antioxidant and antibacterial effects and are expected to be a natural material with whitening, wrinkle improvement, and deodorizing effects for the elderly [17,18,19].

### 3.4. Polyphenol and Flavonoid LC-MS Quantitative Analysis Results

The total polyphenol content in the concentrated extract of guava leaves was measured three times using gallic acid as a standard substance. In addition, the total flavonoid content was measured three times using quercetin as a standard material. The total polyphenol content, converted to gallic acid equivalents, did not show a significant difference based on the ethanol content (* *p* > 0.05). Both the total polyphenol and flavonoid contents, as shown in Table 6, exhibited a trend of decreasing total flavonoid content as the ethanol concentration increased. Flavonoids, as polyphenolic substances, are reported to have differences in solubility in water and ethanol based on their chemical structures [31].

### 3.5. Measurement of Free Radical Erasure Capability (DPPH Method) Results

The results of the DPPH radical scavenging ability measurement of concentrated *P*. *guajava* leaf extracts are shown as follows: Table 7 shows the IC_50_ value, the concentration that eliminates 50% of DPPH radicals. The antioxidant capacities of concentrated *P*. *guajava* leaf extracts were slightly lower than the antioxidant capacity of ascorbic acid used as a positive control. The higher the ethanol content, the higher the antioxidant capacity. These results are consistent with the reported correlation that the higher the content of phenolic compounds such as quercetin, gallic acid, chlorogenic acid, and ferulic acid, the higher the radical scavenging activity [12,32]. In addition, as shown in Table 5 and Table 6, although the flavonoid content of the 70% ethanol extract was lower than that of the 30% and 50% ethanol extracts, the content of other phenolic compounds in addition to flavonoids was high [33] and is thought to exhibit high antioxidant activity.

### 3.6. Antimicrobial Activity Measurement (Disc Diffusion Method) Results

**Figure 2:**
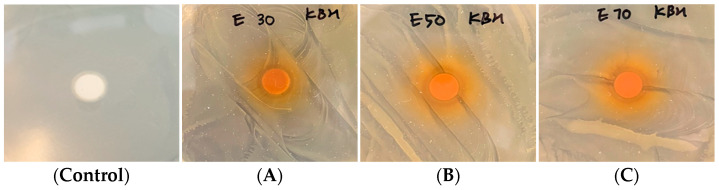
Photo results of *Psidium guajava* leaf extract antibacterial tests: (**A**) 30% ethanol extract, (**B**) 50% ethanol extract, (**C**) 70% ethanol extract.

The antimicrobial efficacy analysis results for concentrated *P*. *guajava* leaf extracts are depicted in Figure 2. Clear zones of 3 mm were observed for all extracts compared to cefotaxime and ampicillin, which are positive controls for *S. aureus*, the causative agent of atopic dermatitis as well as food poisoning. No significant variation in clear zone diameter was evident across extracts, irrespective of the ethanol concentration used for extraction. This suggests that the presence of antimicrobial molecules such as 2,4-di-tert-butylphenol, caryophyllene oxide, and γ-muurolene within extracts is likely to be responsible for the observed effects.

### 3.7. Measurement of Ability to Remove Odor of Elderly People (Fehling Reaction) Results

**Figure 3:**
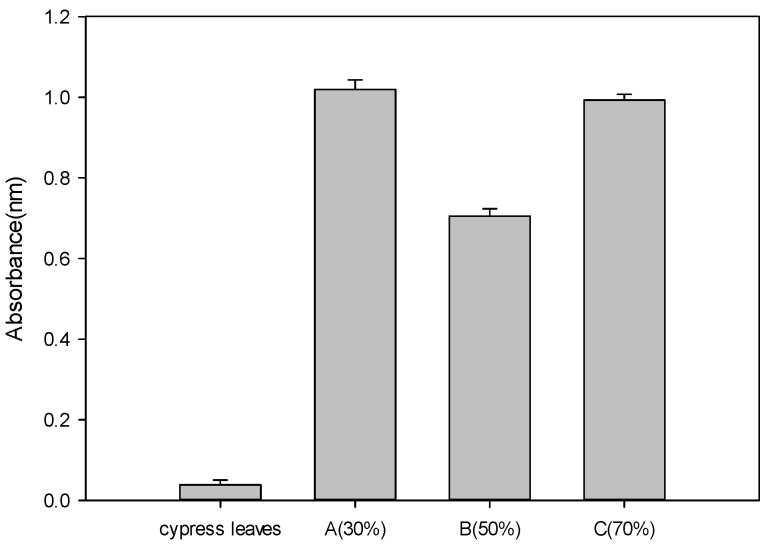
Measurement results of trans-2-nonenal removal activities of *Psidium guajava* leaf extracts and positive control (*Cypress* leaf extract) based on absorbance values: (A) 30% ethanol extract, (B) 50% ethanol extract, (C) 70% ethanol extract (*p* < 0.05).

The results of analyzing trans-2-nonenal inhibition activity of concentrated *P*. *guajava* leaf extract are shown in Figure 3. In the case of the positive control cypress leaf 40% ethanol extract, the result was 97.1 ± 1.24%; in the case of extract A, 31.2 ± 1.78%; in the case of extract B, 52.4 ± 2.45%; in the case of extract C, 33.0 ± 1.96%%. Extract B showed the highest trans-2-nonenal removal rate among extracts, having an inhibitory activity of 52.4 ± 2.45%. However, compared to the positive control group, cypress leaf extract, extract A was 32.1%, extract B was 54.0%, and extract C was 34.0%, showing somewhat insufficient efficacy in removing elderly odor.

### 3.8. Tyrosinase Inhibition Activity Assay Results 

**Figure 4:**
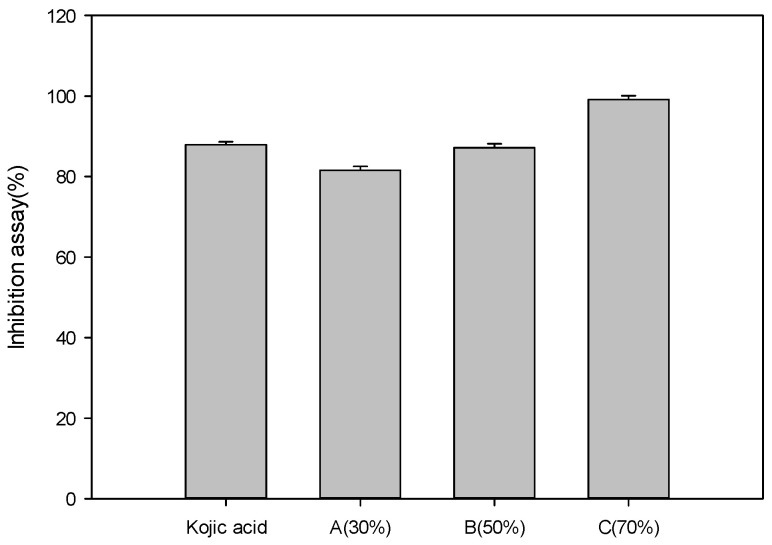
Tyrosinase inhibition activities of concentrated *Psidium guajava* leaf extracts: (A) 30% ethanol extract, (B) 50% ethanol extract, (C) 70% ethanol extract (*p* < 0.05).

The results of analyzing the tyrosinase inhibition activities of concentrated *P*. *guajava* leaf extracts are shown in Figure 4. Kojic acid, the control group, showed an inhibition assay result of 87.9 ± 0.72%. Extract A showed an inhibition assay result of 81.5 ± 1.03%. Extract B showed an inhibition assay result of 87.2 ± 0.98%, and extract C showed a tyrosinase inhibition assay result of 99.1 ± 0.97%. As the ethanol content used for extraction increased, the tyrosinase inhibition assay increased. In particular, the 70% ethanol extract showed a higher whitening effect than the control group of Kojic acid. That is, extract A showed a whitening effect of about 92.7%, extract B about 99.2%, and extract C about 112.7% compared to the positive control group Kojic acid. This was thought to be due to the 1-octadecene molecule in the extract.

### 3.9. Collagenase Inhibition Activity Analysis Results

**Figure 5:**
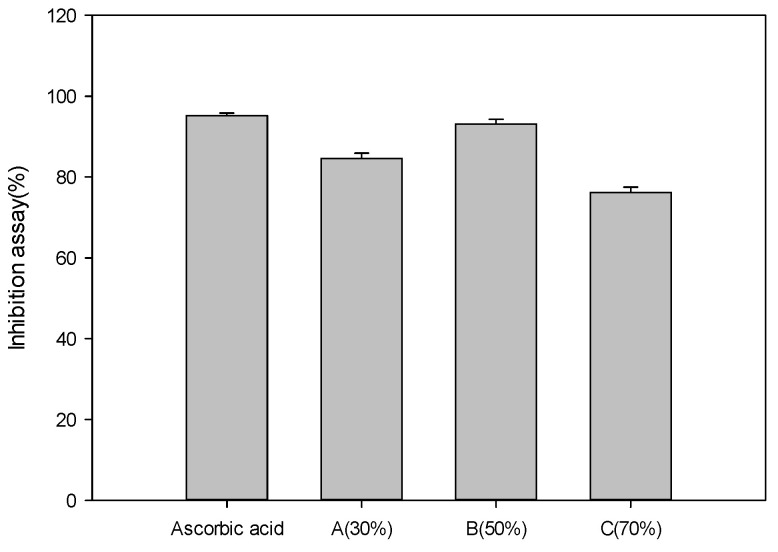
Collagenase inhibition activities of concentrated *Psidium guajava* leaf extracts: (A) 30% ethanol extract, (B) 50% ethanol extract, (C) 70% ethanol extract (*p* < 0.05).

The results of analyzing the collagenase inhibition activities of concentrated *P*. *guajava* leaf extracts are shown in Figure 5. Ascorbic acid, a positive control, showed an inhibitory rate of 95.2 ± 0.64%. Extract A showed an inhibitory rate of 84.6 ± 1.32%. Extract B showed an inhibitory rate of 93.1 ± 1.24%, and extract C showed a collagenase inhibitory rate of 76.2 ± 1.34%. Although the inhibition assay result was lower than that of the control group, the inhibition assay result of extract B was close to that of ascorbic acid, the positive control. Therefore, through this analysis, it was confirmed that the concentrated guava leaf extract had a wrinkle improvement effect of about 88.9% for extract A, about 97.8% for extract B, and 80.0% for extract C compared to ascorbic acid.

## 4. Conclusions

This study studied the antioxidant effect, tyrosinase inhibitory activity, trans-2-nonenal inhibitory activity, and collagenase inhibitory activity through DPPH radical scavenging activity to confirm the possibility of using guava leaf extract concentrate in the field of functional cosmetics and food materials. As a result of the experiment, the DPPH radical scavenging activity of the guava leaf extract concentrate was 90.7% at 5 mg/mL in the case of the 70% ethanol extract. Additionally, in vitro, 70% ethanol showed a higher inhibitory effect on tyrosinase activity than the control Kojic acid, and trans-2-nonenal showed an inhibitory activity of 52.4% in 50% ethanol. These results show that guava leaf extract concentrate has an antioxidant effect due to the radical scavenging activity of phenolic compounds such as sesquiterpenes; has a high content of polyphenols and flavonoids, which are indicators of antioxidant activity; and inhibits collagenase. Because it is active, it has been confirmed that it can be used in functional cosmetics and health functional food materials as a natural extract material.

## Figures and Tables

**Table 1:** The yields of concentrated *Psidium guajava* leaf extracts.

Sample Name	Water and Ethanol (%)		After Decompression (g)	After Enrichment (g)	Yield (%)
A	70	30	312	112	35.8 ± 1.63 ^a^
B	50	50	356	91.9	25.8 ± 2.54 ^b^
C	30	70	443	75.9	17.1 ± 1.54 ^c^

Values are mean ± standard deviation (*n* = 3). Values with different letters within the same column (a–c) differ significantly (*p* < 0.05).

**Table 2:** Colorimetric results of concentrated *Psidium guajava* leaf extract.

No.	A (30% Ethanol Extract)	B (50% Ethanol Extract)	C (70% Ethanol Extract)
L* (Brightness)	20.9 ± 0.56 ^a^	17.7 ± 0.15 ^b^	11.8 ± 0.25 ^c^
a* (Redness)	11.8 ± 0.40 ^e^	8.41 ± 0.06 ^f^	3.20 ± 0.15 ^h^
b* (Yellowness)	14.5 ± 0.25 ^d^	8.41 ± 0.08 ^g^	2.12 ± 0.09 ^i^

Values are mean ± standard deviation (*n* = 3). Values with different letters within the same column (a–i) differ significantly (*p* < 0.05).

**Table 3:** GC-MS chromatogram profile of 30% ethanol extract of concentrated *Psidium guajava* leaves.

Retention Time (RT)	Name of the Compound	Area Peak (%)
5.190	Octane	10.1
6.147	2,4-Dimethyl-1-heptene	4.45
22.97	1-Octadecene	5.76
29.22	1-Docosene	4.49

**Table 4:** GC-MS chromatogram profile of 50% ethanol extract of concentrated *Psidium guajava* leaves.

Retention Time (RT)	Name of the Compound	Area Peak (%)
5.195	Octane	4.79
14.80	Benzene	1.32
17.72	Caryophyllene	1.03
19.03	2,4-Di-tert-butylphenol	1.13
20.27	Caryophyllene oxide	2.61
22.97	1-Octadecene	4.09
29.22	1-Docosene	4.16

**Table 5:** GC-MS chromatogram profile of 70% ethanol extract of concentrated *Psidium guajava* leaves.

Retention Time (RT)	Name of the Compound	Area Peak (%)
5.194	Octane	6.49
6.149	2,4-Dimethyl-1-heptene	2.94
17.72	Caryophyllene	1.18
20.27	Caryophyllene oxide	2.04
21.07	γ-Muurolene	1.34
21.13	Copaene	1.98
22.97	1-Octadecene	4.94
29.22	1-Docosene	4.32

**Table 6:** Total polyphenol content (TPC) and total flavonoid content (TFC) of *Psidium guajava* leaf extract at different ethanol ratios.

Sample	TPC (μg/mL)	TFC (mg GAE/mL)
A (30% ethanol extract)	122.1 ± 10.5 ^b^	2.276 ± 1.43 ^d^
B (50% ethanol extract)	127.6 ± 12.6 ^a^	1.944 ± 0.95 ^e^
C (70% ethanol extract)	106.1 ± 6.87 ^c^	1.461 ± 0.86 ^f^

GAE: gallic acid equivalents. Values are mean ± standard deviation (*n* = 3). Values with different letters within the same column (a–f) are not significantly different (*p* > 0.05).

**Table 7:** IC_50_ for DPPH antiradical capacity of extracts.

No.	IC_50_ (mg/mL)
Control (Ascorbic acid)	0.01 ± 0.00 ^a^
A (30% ethanol extract)	2.70 ± 0.03 ^d^
B (50% ethanol extract)	1.80 ± 0.01 ^c^
C (70% ethanol extract)	1.40 ± 0.01 ^b^

Inhibitory activity was expressed as the mean of 50% inhibitory concentration of triplicate determinations, obtained by interpolation of concentration inhibition curve gallic acid equivalents. Values are mean ± standard deviation (*n* = 3). Values with different letters within the same column (a–d) differ significantly (*p* < 0.05).

## Data Availability

Data are contained within the article.
